# Trend of Suicide Rates According to Urbanity among Adolescents by Gender and Suicide Method in Korea, 1997–2012

**DOI:** 10.3390/ijerph120505129

**Published:** 2015-05-13

**Authors:** Kyung-Hwa Choi, Dong-Hyun Kim

**Affiliations:** 1 Hallym Research Institute of Clinical Epidemiology, Hallym University, 1 Hallymdaehak-gil, Chuncheon, Gangwon-do 200-702, Korea; E-Mail: rosach72@hallym.ac.kr; 2 Department of Social and Preventive Medicine, College of Medicine, Hallym University, 1 Hallymdaehak-gil, Chuncheon, Gangwon-do 200-702, Korea

**Keywords:** suicide trend, suicide method, Korean adolescents, urbanity, annual percent change, average annual percent change

## Abstract

This study aims to quantifiably evaluate the trend of the suicide rate among Korean adolescents from 1997 to 2012 according to urbanity. We used national death certificates and registration population data by administrative district for 15–19 years-old adolescents. The annual percent change (APC) and average annual percent change (AAPC) were estimated by the Joinpoint Regression Program. The suicide rate in the rural areas was higher than that in the urban areas in both genders (males (/100,000), 12.2 *vs*. 8.5; females (/100,000), 10.2 *vs*. 7.4 in 2012). However, the trend significantly increased only in the urban area (AAPC [95% CI]: males 2.6 [0.7, 4.6], females 3.3 [1.4, 5.2]). In urban areas, the suicide rate by jumping significantly increased in both genders (AAPC [95% CI]: males, 6.7 [4.3, 9.1]; females, 4.5 [3.0, 6.1]). In rural areas, the rate by self-poisoning significantly decreased by 7.9% per year for males (95% CI: −12.5, −3.0) and the rate by hanging significantly increased by 10.1% per year for females (95% CI: 2.6, 18.2). The trend and methods of suicide differ according to urbanity; therefore, a suicide prevention policy based on urbanity needs to be established for adolescents in Korea.

## 1. Introduction

The WHO reported that suicide is one of the top causes of death in adolescents, along with road traffic injuries and HIV/AIDS [1]. Suicide has been the leading cause of death among Korean adolescents aged 15–19 years since 2009 [2]. The rates increased 1.4 times in 2012 (males: 8.8/100,000; females: 7.7/100,000) compared with those in 2002 (males: 6.5/100,000; females: 5.4/100,000) [3]. The rate was ranked 18th during the period 2000–2004 (Korea: 6.5, OECD: 7.7 per 100,000), and became ninth during the period 2005–2009 (Korea: 8.2, OECD: 6.5 per 100,000) among OECD countries [4].

Previous studies reported that the suicide rate might depend on the degree of urbanity [5,6,7,8,9,10,11,12,13,14,15,16,17]. The reports on the difference in the suicide rates between the rural and urban areas are controversial. Some studies showed higher rates in rural areas [5,13,15,17], but not all [8,14,16]. Some results were different according to gender and study period [10,12,14,16]. Also, method-specific suicide rates were different according to urbanity [5,11].

Studies on suicide among Korean youths have reported that social demographic factors [12,18], mental health factors such as depression, anxiety disorders, ADHD, alcohol and drug abuse [18,19,20], and family-related factors [18,19,21] were related with suicide. However, recently, stress on academic performance [22] and bullying [23] have become more serious in urban areas than in rural areas in Korea.

Therefore, this study aims to evaluate quantifiably the trend of the suicide rate according to urbanity among Korean adolescents from 1997 to 2012.

## 2. Materials and Methods

### 2.1. Study Subjects

We used national death certificate and registration population data to calculate the suicide rates [24]. By law in Korea [25], a report of death shall be filed for a death certificate such as name, sex, original domicile and citizen personal identification number of the deceased person, and date and place of death. In Korea, the death registration rates are nearly complete, and of all deaths registered, 75.3% in 2002 and 98.3% in 2012 were confirmed by a physician’s diagnosis [3]. Information on the overall suicide rate since 1983 was obtained from the Korean Statistical Information Service (KOSIS), but urbanity-specific rates among adolescents have been available since 1997 [26].

### 2.2. Urbanity

In 1997, Korea had 86 cities (total population: 40 million; 15–19 years old: 3.5 million) and 91 rural areas (total population: 6.5 million; 15–19 years old: 0.5 million). In 2012, Korea had 86 cities (total population: 47 million; 15–19 years old: 3.3 million) and 79 rural areas (total population: 4 million; 15–19 years old: 0.2 million). All areas were classified annually by two types because some rural areas became cities or combined with near cities during the study period of 1997 to 2012 [26].

### 2.3. Suicide Rate and Suicide Method

The tenth revision of the International Classification of Diseases (ICD) codes was used to extract suicide data (X60–X84). The suicide methods of Korean adolescents were classified into the following three major categories according to a report by the Ministry of Health and Welfare [19]: jumping from a high place (X80.0–X80.9), including a house (X80.0), apartment (X80.1), school (X80.2), sports and athletics area (X80.3), street and highway (X80.4), trade and service area (X80.5), industrial and construction area (X80.6), farm (X80.7), and other or unknown places (X80.8–X80.9); self-poisoning (X60.0–X69.9) by consumption of substances such as medication (X60.0–X64.9), alcohol (X65.0–X65.9), organic solvent (X66.0–X66.9), gas (X67.0–X67.9), pesticides (X68.0–X68.9), and other chemicals (X69.0–X69.9); and hanging (X70.0–X70.9). The number of suicide deaths and method-specific suicide rates among adolescents (15–19 years old) according to urbanity were calculated using the national death certificates [27] and registration population in each area [26]. The standard errors of the suicide rates were calculated using a formula with the suicide rate divided by the square root of the number of deaths [28]. To identify the suicide rate trend, the annual percent change (APC) and average annual percent change (AAPC) were estimated by Equations (1) and (2):

*APC_i_ = [Exp(β_i_)-1] × 100* (1)

*AAPC = [Exp(∑W_i_ β_i_ /∑W_i_) − 1] × 100, W_i_ = number of years in the same slope (β_i_)* (2)

When the number of joinpoints is 0, the AAPC is the same as the APC.

### 2.4. Statistical Analysis

A model selection and AAPC comparison test were performed using the Monte Carlo permutation method as minimization of type I error [29]. All analyses were performed using the Joinpoint Regression Program version 4.1.0 [30]. The Joinpoint Regression Program software was developed by the National Cancer Institute in the USA to identify and estimate mortality trends. This is a log-linear, Poisson regression that applies the Monte Carlo permutation test to identify the points where the trend line changes significantly. The analysis starts with the minimum number of joinpoints, a zero point, which is a straight line and tests whether one or more joinpoints are significant and should be added to the model; a maximum of four joinpoints are allowed. In the final model, each joinpoint indicates a significant change in the slope. A zero joinpoint implies that the slope has not changed significantly. The permutation test, which estimates the optimal number of joinpoints, was applied after all analyses at a significance level of 0.05 [30].

## 3. Results

### 3.1. Suicide and Death from all Causes by Gender

Table 1 shows the deaths from all causes and suicide among adolescents by gender. The suicide rate was 9.0 per 100,000 among males and 4.3 per 100,000 among females in 1983, and became 8.8 and 7.6 in 2012, respectively. The proportions of suicide out of all deaths tended to increase in both genders, 5.2% in 1983 to 27.0% in 2012 among males and 4.2% to 42.0% among females, respectively. Male-to-female rate ratio (M/F rate ratio) decreased from 2.1 in 1983 to 1.2 in 2012 (see Table 1).

**Table 1:** Suicide death and death from all causes among adolescents (15–19 years of age) by gender in Korea, 1983–2012.

	Male (15–19 years old)			Female (15–19 years old)			Suicide Rate Ratio (Male/Female)
Calendar Year	All Death (/100,000)	Suicide Deaths (/100,000)	Proportion of Suicides out of all Cause (%)	All Deaths (/100,000)	Suicide Deaths (/100,000)	Proportion of Suicides out of all Cause (%)	
1983	173.0	9.0	5.2	102.3	4.3	4.2	2.1
1984	160.3	9.2	5.7	91.3	4.8	5.3	1.9
1985	153.5	10.5	6.8	86.6	5.1	5.9	2.1
1986	145.4	9.8	6.7	80.6	4.7	5.8	2.1
1987	140.1	7.5	5.4	69.4	3.5	5.0	2.1
1988	132.7	8.6	6.5	64.2	4.7	7.3	1.8
1989	126.1	7.5	5.9	56.8	4.3	7.6	1.7
1990	121.7	8.4	6.9	53.9	3.9	7.2	2.2
1991	122.4	7.6	6.2	47.1	3.4	7.2	2.2
1992	126.4	9.5	7.5	50.9	4.7	9.2	2.0
1993	113.7	9.8	8.6	42.8	5.0	11.7	2.0
1994	113.4	8.5	7.5	47.0	4.6	9.8	1.8
1995	108.0	9.2	8.5	50.2	5.8	11.6	1.6
1996	107.6	12.1	11.2	49.2	8.6	17.5	1.4
1997	100.8	8.3	8.2	41.5	6.7	16.1	1.2
1998	81.5	10.7	13.1	40.2	8.5	21.1	1.3
1999	70.0	8.8	12.6	33.7	6.5	19.3	1.4
2000	61.3	7.0	11.4	30.6	5.6	18.3	1.3
2001	54.1	5.8	10.7	27.6	4.9	17.8	1.2
2002	46.9	6.6	14.1	24.2	5.2	21.5	1.3
2003	49.9	9.9	19.8	25.7	6.3	24.5	1.6
2004	42.6	7.7	18.1	20.2	5.2	25.7	1.5
2005	39.4	7.8	19.8	21.9	7.4	33.8	1.1
2006	38.5	6.6	17.1	20.5	5.9	28.8	1.1
2007	43.3	8.3	19.2	20.3	7.4	36.5	1.1
2008	38.8	8.7	22.4	19.9	7.2	36.2	1.2
2009	38.7	11.5	29.7	22.5	9.8	43.6	1.2
2010	37.7	8.8	23.3	20.0	7.7	38.5	1.1
2011	37.1	10.1	27.2	18.3	7.6	41.5	1.3
2012	32.6	8.8	27.0	18.1	7.6	42.0	1.2

Mortality data 1983–2012, Korean Statistical Information Service (KOSIS) [2].

**Figure 1:**
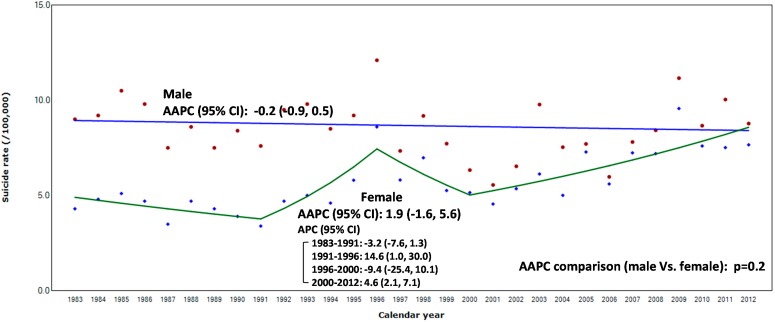
Suicide rate, annual percent change (APC), and annual average percent change (AAPC) according to gender among adolescents,15–19 years of age in Korea, 1983–2012 (data from KOSIS [2]); Model selection and *p*-value of AAPC comparison test estimated using Monte Carlo Permutation method [29].

### 3.2. Suicide Rate by Gender

Figure 1 shows the suicide rate trend according to gender from 1983 to 2012. The rates remained stable between both genders (AAPC [95% CI], male: −0.2 [−0.9, 0.5]; female: 1.9 [−1.6, 5.6]). The period-specific trend (APC) changed among females only (APC [95% CI], 1983–1991: −3.2 [−7.6, 1.3], 1991–1996: 14.6 [1.0, 30.0], 1996–2000: −9.4 [−25.4, 10.1], 2000–2012: 4.6 [2.1, 7.1]), but not among males. When comparing the AAPCs between genders, they were not significantly different (*p* = 0.2).

### 3.3. Suicide Rate by Urbanity

**Figure 2:**
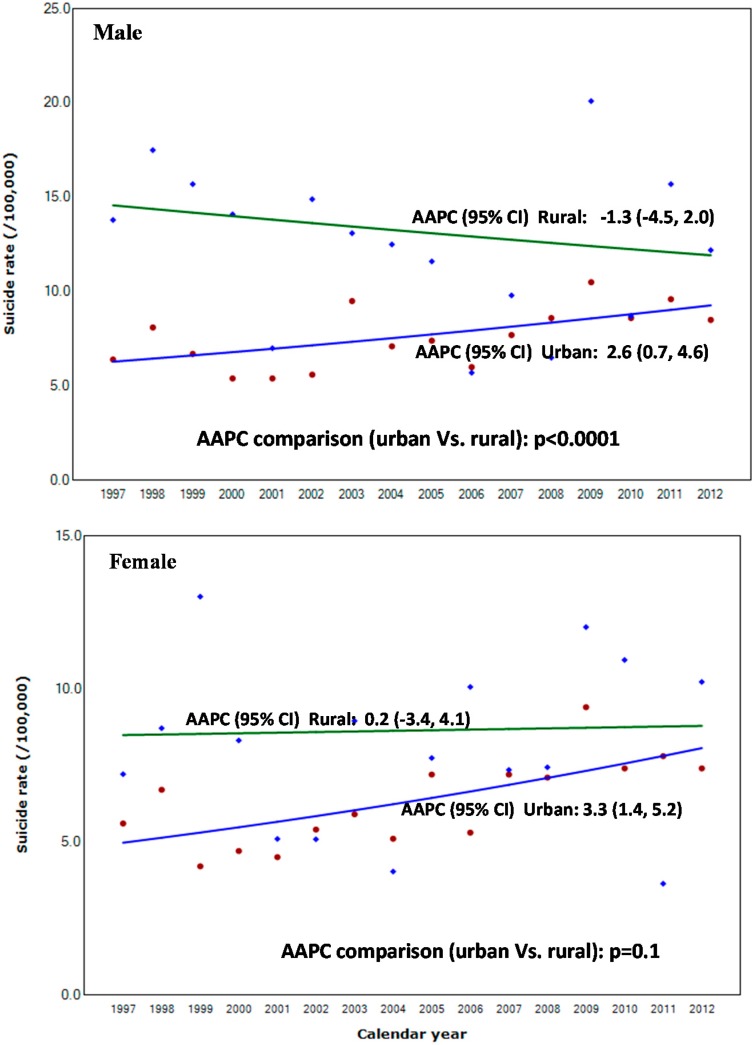
Suicide rate and annual average percent change (AAPC) according to urbanity among adolescents, 15–19 years of age by gender in Korea, 1997–2012; Model selection and *p*-value of AAPC comparison test estimated using Monte Carlo Permutation method [29].

Figure 2 shows the suicide rate trend from 1997 to 2012 according to urbanity. Among males, the urban rate significantly increased by 2.6% per year [95% CI: 0.7, 4.6], while the rate in the rural areas decreased 1.3% per year [95% CI: −4.5, 2.0]. 

When comparing the AAPC between the urban and rural areas among males, the trend was significantly different (*p* < 0.001). Among females, the rate in urban areas significantly increased 3.3% annually [95% CI: 1.4, 5.2], while the rate in the rural areas increased 0.2% annually [95% CI: −3.4, 4.1]. When comparing the AAPC between the two areas among females, the trend was not significantly different (*p* = 0.1).

**Figure 3:**
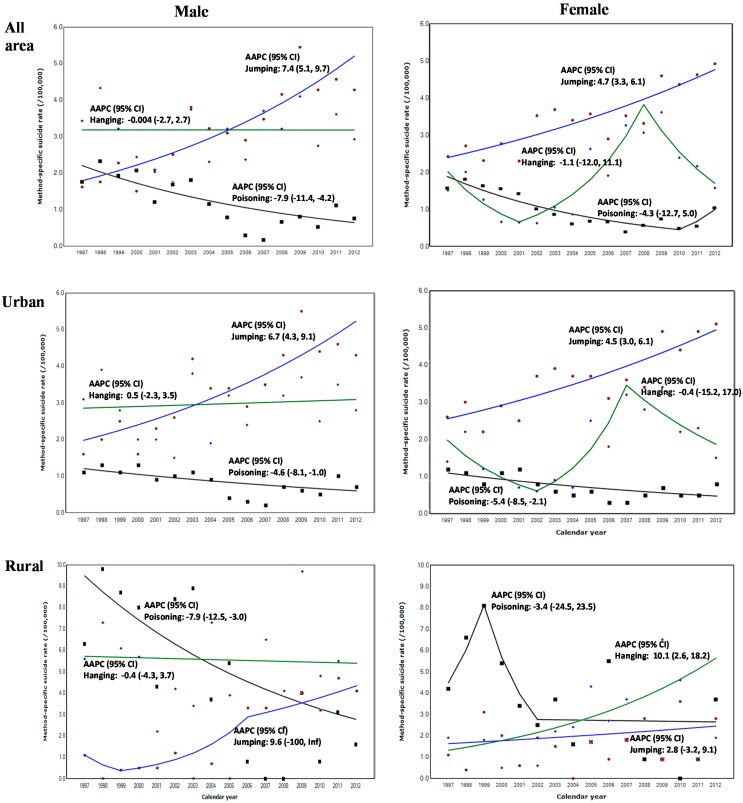
Method-specific suicide rate and annual average percent change (AAPC) according to urbanity among adolescents, 15–19 years of age by gender in Korea, 1997–2012; Model selection using Monte Carlo Permutation method [29].

### 3.4. Method-Specific Suicide Rate by Urbanity

Figure 3 shows the method-specific suicide rates by gender from 1997 to 2012 according to urbanity. For all areas, the suicide rate from jumping significantly increased in both genders (AAPC [95% CI]: male, 7.4 [5.1, 9.7]; females, 4.7 [3.3, 6.1]), while the rate from self-poisoning significantly decreased by 7.9% per year only for males [95% CI: −11.4, −4.2].

In urban areas, the suicide rate from jumping significantly increased in both genders (AAPC [95% CI]: males, 6.7 [4.3, 9.1]; females, 4.5 [3.0, 6.1]), while the rate from self-poisoning significantly decreased between both genders (AAPC [95% CI]: males, −4.6 [−8.1, −1.0]; females, −5.4 [−8.5, −2.1]). In rural areas, the suicide rate from self-poisoning significantly decreased 7.9% per year only for males (95% CI: −12.5, −3.0), while the rate from hanging significantly increased 10.1% per year only for females (95% CI: 2.6, 18.2).

## 4. Discussion

The suicide rate for males was higher than for females, but the M/F rate ratio of suicide tended to decrease steadily from 1983 to 2012 in Korean adolescents. When classified according to urbanity, the suicide rate in the rural areas has been higher than in the urban areas, but the trend significantly increased only in the urban areas.

In this study, the suicide rate was calculated using the Korean national death data. Of all the deaths registered, 75.3% in 2002 and 98.3% in 2012 were confirmed by a physician’s diagnosis [3]. In a previous study that examined the deaths that occurred from 2006 to 2008 in Korea, Cheong *et al.* investigated the suicide rates between urban and rural areas among all ages [12]. Cheong *et al.*’s study calculated 3-year suicide rate and classified the areas into two types of administrative areas [12], which was also done in the present study. Additionally, similar to the present study, studies conducted in Taiwan [5], Iran [8], the USA [31], Australia [10,32], Belarus [16], and UK [6], and one comparing the suicide rates in Beijing (China) and Australia [14] used national data. However, in some studies conducted in India [15] and China [9,13,17], the rates were estimated using sampling data.

### 4.1. Suicide Rate by Urbanity

In the previous study from 2006 to 2008, the suicide rate among males in the urban areas was higher than the rate in the rural areas (urban, 2.6/100,000; rural, 2.2/100,000) and the results for females were opposite (urban, 2.2/100,000; rural, 2.5/100,000) in Korean adolescents 19 years or younger [12]. The study investigated only a certain period. The suicide rate for both genders in the rural areas was higher than the rate in the urban areas in China [13,14], and USA [31]. Moreover, the suicide rates in the USA was observed to increase with increasing rurality [31].

The rate among males in the rural areas was higher than that in the urban areas, but the results for females varied according to the time period in the Australia [10,14] and Belarus [16] studies among adolescents aged 15–24 years. Therefore, we suggest that studying the suicide rates for a period as long as possible in a country is needed and important because the pattern could vary according to the study period.

The results of a comparative study on suicide rates in urban and rural areas, conducted among all ages, were not different from those conducted on adolescents [5,12,13,14,15,16,17]. However, the rate in the urban areas was higher than in the rural areas in a study conducted in southwestern Iran from 2004 to 2009 [8].

The causes of the higher rural suicide rate can be classified into deprivation factors and “others”. Deprivation factors include difficulties in accessing health care, lower utilization, lower SES [6,11,33], and social welfare expenditure level [12]. “Others” are the social characteristics such as isolation, strict rural life, and male-dominated culture of rural areas only [10] and depression, which is one of the suicide risk factors and might not be treated because the person cannot find a clinic to diagnose the depression [12]. Suicide-related deprivation factors were more significant in urban areas than in rural areas in an Australian ecological study from 2004 to 2008 among 15–59 years old people [34]. This means that suicide-related deprivation factors such as the unemployment rate and low-skilled jobs in urban areas, may not be risk factors in rural areas [34]. Rural areas appear to have regions with different characteristics: garden suburbs with high SES, areas with advanced agriculture and high income, and poor farms [6]. Further research is needed to identify the cause of the higher adolescent suicide rate in rural areas.

### 4.2. Suicide Rate Trend by Urbanity

Similar to the findings of the study conducted on adolescents aged 15–24 years (4.0 in 1988 and 7.2 in 1997) in Australia from 1988 to 1997, in the present study, the suicide rate was observed to increase only in the urban areas, especially in females [32]. Additionally, similar to the findings of the present study, although a different age groups was studies, and the study period was only five years, the suicide rate has also been found to increase in the urban areas in Iran [8]. However, the suicide rate in the rural areas was found to have increased more than that in the urban areas in an English study conducted on adolescents aged 15–24 years from 1981 to 1998 (Relative risk of increasing rurality: male, 1.16; female, 1.28) [6].

There is no study that compares the suicide rate trend between the Korean rural and urban areas, but Cheong *et al.* [12] reported that the suicide rate is high among the urban poor in urban areas where the gap between the rich and the poor is bigger than it is in the rural areas. In this study, we suggest the reason for the increase in the suicide rate among urban adolescents. We observed that the suicide methods differed in the urban and rural areas. The suicide rate by jumping, which is the most lethal method, only increased in urban areas because of the higher concentration of high buildings in urban than in rural areas. In Korea, in 2010, 66.5% of the houses in urban areas were apartments, while it was 32.3% in rural areas [35]. This implies that the housing environment could be a cause of increasing suicide rates by jumping in urban areas.

In addition, the development of an emergency care and transportation system in recent decades may have dramatically reduced the success rate of suicide attempts from self-poisoning, which was one of the most common methods of committing suicide in the rural areas [9].

### 4.3. Suicide Rate by Gender

In this study, the M/F rate ratio of suicide decreased in Korean adolescents during the study period from 1983 to 2012 because the suicide rate has increased since 2000 among females only but not among males. The rate ratio, which was reported to be 1.2 in 2012, was lower than that observed in other countries. WHO data on 90 countries revealed that the rate ratio for adolescents aged 15–19 years was 2.6 on an average [36]. Even in all ages, the rate ratio was lower in Korea [2] than in OECD countries [37]. *Philips et al.*, reported that Asian young women are high-risk group for suicidal behavior and the trend over the past several years has been in the opposite direction: rates in women have been stable or decreasing while rates in men have been increasing particularly among young age-groups [13]. It might be interpreted that there is a difference between the M/F ratio of suicide western countries and Korea for that reason. The proportion of suicide attempts and suicidal ideation among female adolescents was reported to be higher than that among male adolescents in Korea [38] because, in combination with depressive symptoms, reaction to external stress, such as the academic stress of entering university, bullying at school, or family violence, might have a higher on suicidal behavior in female adolescents than in male adolescents in Korea [39]. The risk of suicide attempts could be increasing among those who have already attempted suicide [40]. Also, the results in this study imply that increasing lethal method *i.e.*, jumping and hanging caused increasing suicide rates among adolescents, especially among females.

### 4.4. Suicide Method

Differences in suicide methods used in urban and rural areas was also observed in other countries such as China [9], Taiwan [5], and Australia [32]. In Shandong, China, pesticide ingestion was the most common suicide method in rural areas, while hanging was the most frequently used method in urban areas from 2004 to 2010 among all ages [9]. However, it is difficult to compare these results with the findings of the present study because different age groups were studied. Similar to the findings of the present study, the suicide rate by jumping has been increasing, and the rate by poisoning has been decreasing in the 24 years or younger group in Taiwan [5]. Further, in Australia, in 1988, the most frequent methods were hanging among males and poisoning among females in metropolitan areas, and firearms among both genders in non-metropolitan areas [32]. Firearms are not a common method in Korea because it is not legal to possess firearms without permission [41]. The suicide rate by hanging has been increasing in Australian adolescents [32]. However, the same was observed only in rural females in the present study. In India, poisoning was the most common method followed by hanging [15]. However, it is difficult to compare these findings with those of the present study because the specific suicide rates were estimated without considering urbanity and age group.

### 4.5. Limitations and Strength

The obvious cause of suicides among adolescents was not investigated in this study. However, the causes of suicides might be expected to vary by time and region because the trend of suicide rate was different according to urbanity and period in this study. The suicidal risk factors between rural and urban area were different in previous studies [12,34,42], but there has been little research in the difference between the suicidal risk factors according to period. Further research will be needed for this.

It is difficult to define rural areas because rural areas are being rapidly urbanized in Korea. Hence, this study classified annually rural and urban areas from 1997 to 2012 because some rural areas became administratively urban areas. When classified according to urbanity based on administrative area in 1997, the results were not different with those of the present study (Data not shown). The change was not big because only seven among the 91 rural areas became administratively urban areas during the study period. Despite these limitations, the present study is the first to examine the trend in the suicide rates according to urbanity in Korean adolescents quantifiably.

## 5. Conclusions

The suicide rate among Korean adolescents in the rural areas was higher than in the urban areas. However, the suicide rate only in the urban areas has consistently increased in Korea, especially for females. The Korean government has established and operated suicide prevention center and mental health center for the general population since 2011 [43]. It plans to build a foundation for youth suicide prevention in 2015–2016, to develop a suicide prevention program, to strengthen the early diagnosis and treatment system, and to operate a youth suicide prevention support system in 2017 [43]. However, the on-going youth suicide prevention policy in Korea does not consider urbanity. The Korean government should establish a suicide prevention policy for adolescents according to urbanity.
